# Prevalence of metabolic abnormalities in patients with urolithiasis in Kinshasa, Democratic Republic of Congo

**DOI:** 10.11604/pamj.2021.40.75.28349

**Published:** 2021-10-05

**Authors:** Pablo Kuntima Diasiama Diangienda, Dieudonné Molamba Moningo, Ernest Kiswaya Sumaili, Alain Ngoma Mayindu, Augustin Monga Lembe Punga-Maole, Jean-Philippe Haymann, Michel Daudon

**Affiliations:** 1 Department of Urology, University Hospital of Kinshasa, Kinshasa, Democratic Republic of Congo,; 2 Department of Nephrology, University Hospital of Kinshasa, Kinshasa, Democratic Republic of Congo,; 3 Department of Clinical Biology, University Hospital of Kinshasa, Kinshasa, Democratic Republic of Congo,; 4 Department of Functional Investigations, Tenon Hospital, Paris, France

**Keywords:** Urolithiasis, insufficient diuresis, hypocitraturia, composition

## Abstract

**Introduction:**

metabolic abnormalities are key factors in urolithiasis patients because they can be modified to prevent the risk of urinary stones. The objectives of this study were to estimate the frequency of metabolic abnormalities in the urine of patients with urolithiasis and to determine their possible link with the chemical composition of stones.

**Methods:**

we conducted a cross-sectional study evaluating 73 patients referred for urolithiasis in 8 clinics in Kinshasa, between January 2017 and September 2019. Twenty four-hour or early morning urine were collected and analyzed in the Tenon Hospital in Paris. Parameters analyzed included pH, specific gravity, creatinine, uric acid, calcium, phosphate, oxalate, citrate and magnesium. Chi square test or chi-square likelihood-ratio and student's t test were used as statistical tests.

**Results:**

overall, 89% (n=65) of patients with lithiasis had metabolic abnormalities. Mean (SD) age of patients was 47.0 (14.2) years with male to female ratio of 1.6: 1. The mean (SD) 24-hour diuresis was 1836.4 (1216.9) ml; the mean (SD) urine density was 1.018 (0.007); and the mean (SD) pH was 6.1(0.8). Hypocitraturia was the most frequently observed metabolic abnormality and was found in 76.7% patients. Other significant metabolic abnormalities were low magnesuria (35.6%), hyperoxaluria (11%), and low sulphaturia (74%). Whewellite (73.5%) was the main chemical component. The mean pH was higher in patients with carbapatite and struvite stones (p=0.031).

**Conclusion:**

this study suggests that inadequate diuresis and hypocitraturia were important lithogenic factors. The population should be encouraged to increase water intake to limit the frequency of urine super saturation with crystals.

## Introduction

Urolithiasis is one of the most common urinary tract diseases. Its prevalence is growing worldwide. This growth may be caused by shift in diet and lifestyle, higher prevalence of chronic diseases such as obesity and diabetes, global warming, with rising temperature resulting in dehydration and high urinary concentration of calcium and other stone-forming salts. Various factors may be associated to susceptibility to urolithiasis including race, gender, diets, genetics, climatic aspects, and metabolic changes. Indeed, changes in lifestyle and western dietary habits can cause urinary metabolic abnormalities, which represent important risk factors for stone disease. Thus, metabolic evaluation is indicated in all patients with stone formation to identify urolithiasis risk factors with the ultimate goal of preventing recurrence [1,2]. Metabolic evaluation is carried out in 24-hour urine, first-morning urine (collected in the morning on an empty stomach), and/or blood sample depending on the results of the stone analysis [1,2]. Urinary and serum exploration should be performed on an outpatient basis, at least one month from any obstructive episode or urological operation, including extracorporeal lithotripsy (ECL) [1]. Despite the general agreement that a complete metabolic evaluation is indicated in all patients with urolithiasis, the practice is not a standard of care in our hospital for various reasons including the scarcity of well-equipped laboratories and the socio-economic issues. A lack of data on metabolic abnormalities in patients with urolithiasis hampers effective lobbying for the introduction of preventative strategies. To our knowledge, no previous study has been conducted to estimate the prevalence of metabolic abnormalities in patients with urolithiasis in the Democratic Republic of Congo (DRC), but such a study would be beneficial for the development of rational, cost-effective preventive strategies. Thus, to fill this gap, we undertook this study. We also investigated the association between metabolic abnormalities and the chemical composition of stones.

## Methods

**Study design and setting:** from January 2017 to September 2019, we conducted a cross-sectional study including patients with urolithiasis attending eight clinics in Kinshasa.

**Study population:** the study involved patients for the most part easily accessible in hospitals that agreed to collaborate. The sample size was not predetermined in the initial phase of the study and the sample approach was carried out by reasoned or strategic choice; thus, 73 patients had taken part in this study. Only patients with urolithiasis followed in these hospitals (during our study period) with a medical file containing essential parameters for this study and who accepted the 24-hour or early morning urine waking up were included in the study. Patients not meeting these criteria were excluded from the study.

**Data collection:** data from patients with urolithiasis were collected using pre-established forms. Urine collection was performed at least one month after surgical extraction or spontaneous stone removal. In some patients, this sample was taken during the first consultation (at least one month before the stone was removed). Twenty four-hour (n=35) or early morning (n=38) urine was first analyzed with a urine dipstick and acidified with pure hydrochloric acid (1% V/V). Following homogenization, samples in test tubes were stored at 6°C at the University Hospital of Kinshasa for a maximum period of six months before being shipped to the Functional Investigations Department of the Tenon Hospital in Paris.

**Definitions:** urine creatinine was measured by the Jaffé colorimetric method. Calcium and magnesium measured by atomic absorption spectrophotometry, and the determination of oxalate, citrate and sulfate by ion chromatography. The magnesium/calcium and calcium/creatinine ratios were used to evaluate low magnesuria and hypercalciuria in the urine of awakening. For each lithiasic patient another urine sample was taken for urine culture (carried out in Kinshasa). In addition, blood sample was taken from each patient to measure serum creatinine (carried out in Kinshasa). Only stones extracted or eliminated spontaneously during our study period were analyzed by Fourier transform infrared spectrophotometry at the Tenon Hospital (APHP, Paris, France). All stones were first kept dry in vials at the University Hospital of Kinshasa before being sent to Tenon Hospital. The stones analyzed were classified according to their main component (chemical or crystalline body representing the large proportion in a given stone). These main components were grouped according to their chemical family: calcium oxalate (including whewellite and weddellite), carbapatite, struvite and ammonium urate. Patient demographic and clinical data were obtained from medical records and during the medical appointment, including age, sex, place of residence, weight and height to calculate the body mass index (BMI), occupation, site of stones, existence of a urinary tract infection, comorbidities, and serum creatinine. Age was divided into 4 categories: < 20 years, 20-39 years, 40-59 years, and ≥ 60 years. The profession was categorized in 3 groups: civil servant, liberal, and student/pupil and unemployed. Any employee of the state public service was considered a civil servant, while any unemployed individual with or without professional qualification was considered unemployed.

**Statistical analysis:** continuous variables were expressed as means and medians. Categorical variables were summarized into proportions. Differences in categorical variables between groups were assessed using Chi square test or the chi-square likelihood-ratio as appropriate. Differences in means were assessed by the student´s t test. P values less or equal to 0.05 were interpreted as statistically significant. Statistical analysis was performed using SPSS statistics software version 22 (IBM, Armonk, USA).

**Ethical considerations:** this study was approved by the ethics committee of the School of Public Health at the University of Kinshasa (approval number: ESP/CE/29/2020). Participation of human research subjects conformed to institutional review board guidelines, applicable laws and the World Medical Association Declaration of Helsinki.

## Results

**Figure 1:**
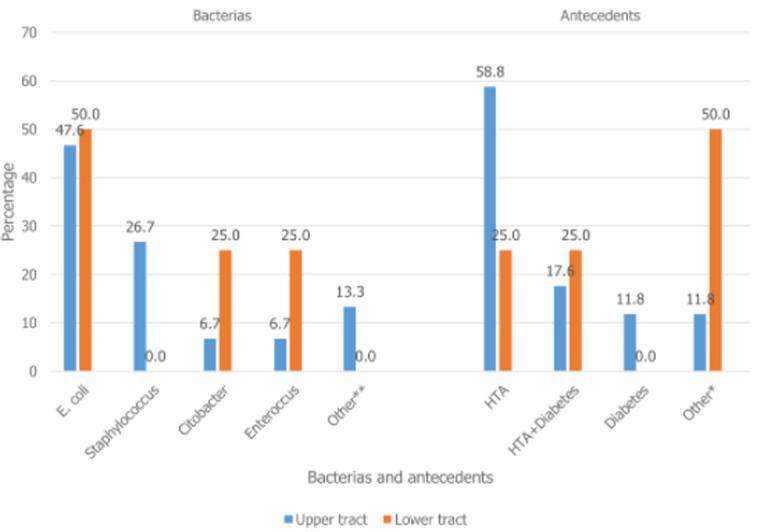
distribution of germs and antecedents according to the site of the stones *pregnancy, single kidney, double j probe and hypertension + Gout, **Acetobacter and Klebsiella

**General characteristics of the population:** the mean (SD) age of the patients was 47.0 (14.2) years. Most patients were males (62%), making a male to female ratio of 1.6: 1. The majority of patients (86.3%) resided in the city of Kinshasa and others lived in the provinces. As depicted in Table 1, 6 patients (8.2%) were undernourished (BMI <18.5kg/m^2^). Urinary tract infections were observed in 19 patients (26.6%). Escherichia coli was the predominant bacteria (Figure 1). Co-morbid factors were identified in 28.8% of patients. Sixteen patients (21.9%) were hypertensive (Figure 1).

**Table 1:** sociodemographic characteristics by site of stones

Variables		Siège des calculs		p
	All n=73 (%)	Upper tract n=57 (%)	Lower tractn=16 (%)	
**Age (years**)				0.415
≤ 19	2(2.7)	1(1.8)	1(6.3)	
20-39	21(28.8)	15(26.3)	6(37.5)	
40-59	35(47.9)	30(52.6)	5(31.3)	
≥ 60	15(20.5)	11(19.3)	4(25.0)	
**Sex**				0.037
Females	28(38.4)	25(43.9)	3(18.8)	
Males	45(61.6)	32(56.1)	13(81.3)	
**Residence**				0.380
Kinshasa	63(86.3)	50(87.7)	13(81.3)	
Outside Kinshasa	10(13.7)	7(12.3)	3(18.8)	
**Profession**				0.925
Unemployed	28(38.4)	21(36.8)	7(43.8)	
**Officia**l	28(38.4)	23(40.4)	5(31.3)	
Liberal	9(12.3)	7(12.3)	2(12.5)	
Student	8(11.0)	6(10.5)	2(12.5)	
**BMI**				0.385
Normal	29(39.7)	21(36.8)	8(50.0)	
Overweight	23(31.5)	18(31.6)	5(31.3)	
Obesity	15(20.5)	14(24.6)	1(6.3)	
Undernutrition	6(8.2)	4(7.0)	2(12.5)	

Bmi; body mass index

**Urinary metabolic abnormalities:** one or more urinary metabolic abnormalities were found in 89% (n=65) of patients. The mean (SD) number of metabolic abnormalities per patient was 2.1 (1.1) with extremes ranging from 0 to 4. The mean (SD) 24-hour urine output was 1836.4 (1216.9) ml and half of patients had a 24-hour urine output less than 1296ml, while the mean (SD) early-morning urine volume was 199.6 (105.5) ml. Overall, the mean (SD) urine density was 1.018 (0.007). No statistically significant difference between the method of urine collection and urine density was observed (1.019 ± 0.007 early-morning vs. 1.018 ± 0.007 24-hour urine, p= 0.748). The general mean (SD) urine pH was 6.1 (0.8). The mean pH of early-morning urine was higher than that of 24-hour urine (6.5 ± 0.7 vs. 5.8 ± 0.9, p < 0.001). There was no statistically significant difference between urine pH and stone site. The mean (SD) protein concentration in the early-morning urine was 0.175 (0.41) g/L with a median of 0.02g/L. The mean (SD) 24 hour proteinuria (n= 35) was 0.145 (0.04) g/ 24 hours and 75% of patients had 24 hour proteinuria less than 0.03g/24 hours.

The mean (SD) concentration of oxalate in the early-morning urine was 0.20 (0.14) mmol/L and its mean (SD) daily excretion was 0.25 (0.18)mmol/24H. The mean (SD) concentration of calcium and its mean (SD) daily excretion was 2.43 (1.86) mmol/L and 2.62 (2.2) mmol/L. The mean (SD) concentration (in mmol/L) and daily excretion (in mmol/24 hours) of citrate, creatinine, magnesium, phosphate, sulphate and urea were respectively 0.87 (1.29) (median of 0.25) and 0.68 (1.38) (75% of patients had a daily citrate excretion of 0.14 mmol/24 hours), 7.93 (5.35) and 4.84 (6.65) (75% of patients had daily creatinine excretion of 8.37), 2.86 (1.94) and 3.28 (2.18), 10.88 (7.69) and 11.85 (8.61), 6.14 (4.36) and 6.40 (4.36), 143.87 (77.43) and 165.70 (104.23). The mean (SD) calcium/creatinine ratio was 1.12 (4.36) mmol/mmol (median of 0.36) and the mean (SD) magnesium/calcium ratio was 1.52 (1.36) mmol/mmol. Proteinuria was more common in patients with lower tract stones (47.8% vs. 17.5%, p=0.035). Nineteen patients (26%) had hypercalciuria, 35.6% had low magnesuria, 11% had hyperoxaluria, 76.7% had hypocitraturia, and 65.7% had low sulphaturia. Only 14.3% (n= 35) of patients had a protein intake within the standards. Low magnesium was more frequent in adults and patients 60 years old or greater (42.9% adults and 53.3% patients over 60 years old, p= 0.027). This association was also observed between low sulphaturia (74.3% adults and 80% patients over 60 years old vs. 47.6% young adults and 0% children, p= 0.039).

**Stone composition and urinary metabolic abnormalities:** only urinary stones from 49 patients (67.1%) were analyzed (the other lithiasic patients did not remove their stones during our study period). The average (SD) number of components per stone was 5.1 (1.6). A single stone of whewellite could be considered pure excluding the protein frame (less than 5%). The whewellite, present predominantly in 36 stones (73.5%), was the most frequent main component. Other main components included carbapatite (8.2%), weddellite (8.2%), struvite (6.1%). and ammonium urate (4.1%). Patients with carbapatite and struvite stones had higher urine pH compared to those with calcium oxalate stones (p = 0.031). Mean protein excretion was higher in carbapatite stones (p = 0.004), (Table 2). Mean (SD) urinary pH was higher in patients with carbapatite and struvite stones (p = 0.031). The mean urinary protein excretion was higher in carbapatite stones (p = 0.004) (Table 2). Struvite was nearly three times more associated with proteinuria compared to calcium oxalate (p = 0.047). Also, all patients with ammonium urate stones and 42.5% of those with calcium oxalate stones had low magnesuria (p = 0.018) (Table 3).

**Table 2:** average concentrations and average daily excretions main components of stones

	Type of stones				p
Variables	CaOx	CA	PAM	UrAm	
Urine pH	6.09±0.83	6.87±0.25	6.67±0.29	5.75±1.06	0.031
**Average concentrations**					
Protein (g/L)	0.13±0.30	0.56±0.62	0.95±1.30	0.54±0.32	0.004
Calcium (mmol/L)	2.61±1.86	2.66±2.24	1.41±0.91	3.90±4.66	0.720
Calcium/creatinine (mmol/mmol)	0.46±0.25	0.35±0.28	0.19±0.18	0.43±0.00	0.389
Magnesium (mmol/L)	3.07±2.13	2.25±1.68	1.41±1.23	3.03±1.40	0.223
Magnesium/calcium (mmol/mmol)	1.33±1.11	1.05±0.52	1.48±0.21	0.56±0.00	0.465
Oxalate (mmol/L)	0.20±0.14	0.20±0.10	0.10±0.03	0.20±0.16	0.336
Citrate (mmol/L)	0.95±1.27	0.67±0.99	0,27±0,35	0,79±1,05	0.898
Sulfate (mmol/L)	5.43±4.09	4.22±2.84	2.77±1.89	7.85±8.56	0.339
**Average daily excretions**					
Proteines (g/24H)	0.08±0.31	0.00±0.00	0.43±0.75	0.13±0.18	0.524
Calcium (mmol/24H)	1.25±1.65	0.00±0.00	2.57±4.46	0.24±0.35	0.340
Magnesium (mmol/24H)	1.70±2.35	0.00±0.00	1.17±2.02	0.82±1.17	0.320
Oxalate (mmol/24H)	0.12±0.19	0.00±0.00	0.10±0.18	0.03±0.05	0.329
Citrate (mmol/24H)	0.40±1.11	0.00±0.00	0.07±0.13	0.02±0.03	0.311
Sulfate (mmol/24H)	3.15±4.04	0.00±0.00	0.97±1.67	0.75±1.06	0.266

CaOx: calcium oxalate, CA: carbapatite, PAM: struvite, UrAm: ammonium urate. *likelihood ratio chi-square

**Table 3:** distribution of metabolic abnormalities by main components

		Type of stones				
Variables	All n=49(%)	CaOx n=40(%)	CA n=4(%)	PAM n=3 (%)	UrAm n=2	p
**Urinaire pH**						0.559
< 6	14(28.6)	13(32.5)	0(0.0)	0(0.0)	1(50.0)	
6-7	32(65.3)	24(60.0)	4(100.0)	3(100.0)	1(50.0)	
> 7	3(6.1)	3(7.5)	0(0.0)	0(0.0)	0(0.0)	
**Proteinuria**						0.047*
Yes	15(30.6)	9(22.5)	2(50.0)	2(66.7)	2(100.0)	
No	34(69.4)	31(77.5)	2(50.0)	1(33.3)	0(0.0)	
**Hypercalciuria**						0.150
Yes	8(16.3)	5(12.5)	2(50.0)	1(33.3)	0(0.0)	
No	41(83.7)	35(87.5)	2(50.0)	2(66.7)	2(100.0)	
**Low Magnesuria**						0.018*
Yes	19(38,8)	17(42.5)	0(0.0)	0(0.0)	2(100.0)	
No	30(61.2)	23(57.5)	4(100.0)	3(100.0)	0(0.0)	
**Hyperoxaluria**						0.161
Yes	5(10.2)	3(7.5)	1(25.0)	0(0.0)	1(50.0)	
No	44(89.8)	37(92.5)	3(75.0)	3(100.0)	1(50.0)	
**Hypocitraturia**						0.817
Yes	36(73.5)	29(72.5)	3(75.0)	3(100.0)	1(50.0)	
No	13(26.5)	11(27.5)	1(25.0)	0(0.0)	1(50.0)	
**Low Sulfaturia**						0.586
Yes	33(67.3)	26(65.0)	3(75.0)	3(100.0)	1(50.0)	
No	16(32.7)	14(35.0)	1(25.0)	0(0.0)	1(50.0)	

CaOx: calcium oxalate, CA: carbapatite, PAM: struvite, UrAm: ammonium urate

## Discussion

In this study, a total of 89% of patients presented with one or more urinary metabolic abnormalities with a mean (SD) abnormalities per patient of 2.1 (1.1). Half of patients had a 24-hour urine output less than 1296 ml, the mean urine density of the whole group was 1.018 ± 0.007, the mean urine pH was 6.1 ± 0.8 and 76.7% had hypocitraturia. Low magnesuria (p = 0.027) and low sulphaturia (p=0.039) were related to patient age. Struvite (as the majority body) was associated with elevated urinary pH and proteinuria, while calcium oxalate was associated with low magnesuria (p = 0.018). The prevalence of urinary metabolic abnormalities in this study (89%) was lower compared to 97.5% found by Yang *et al*. [3] in China. The difference can be partially explained by the study population. The study by Yang *et al*. only included a population aged 0 to 18 years old. Like data from previous studies, our findings suggest that inadequate hydration might contribute to lithiasis. In fact, low diuresis promotes an increase in the concentration of lithogenic solutes capable of worsening an imbalance between promoters and inhibitors of urinary crystallization [4]. Although there are various theories involved in lithogenesis, the super saturation of the urinary environment remains one of the key factors in the formation of urinary stones [5]. For this reason, Traxer *et al*. [2] recommends 24-hour urine collection as a trick to make patients aware of the effort required to achieve a recommended urine output of two liters per 24 hours. In this study, more than half of the patients had a 24-hour urine output of less than 1500ml. Yang *et al*. [3] found in their study that only 12.5% of patients had small urine volumes and higher urine volumes were recorded in girls than in boys. The DRC is a resource-limited country. Thus, access to clean water is extremely low. In addition, the DRC is a country with a tropical climate and temperature is high nearly all year, which can cause of dehydration.

In this study, hypocitraturie (76.7%) was the predominant inhibitor of crystallization. This is in line with findings, by Yang *et al*. [3] and Kovacevic *et al*. [6] who found that hypocitraturia was the common metabolic abnormality in their study populations with a respective frequency of 97.5% and 58.1%. Kovacevic *et al*. [6] also found that urinary citrate was positively correlated with urinary potassium and urinary magnesium. Indeed, several substances are considered as inhibitors of crystallization in particular, ions and small molecules; medium size molecules and macromolecules [2,7-14]. Three substances, including the Tamm-Horsfall protein, nephrocalcin, and citrate ions appear to have more influence over the others regarding oxalo-calcium crystallization [5]. Thus, Wattanachai *et al*. [15], found that potassium citrate supplementation significantly increased urinary pH and urinary potassium citrate excretion. Taken together, these data suggest that citrate salt can be used as a treatment option in patients with urolithiasis. Citrate can be found in various fruits and vegetables, but its main source is endogenous and urinary excretion of citrate is essentially dependent on the intracellular acid-base balance. Any situation that generates acidosis will tend to increase tubular reabsorption of citrate and lead to hypocitraturia [16-18].

Among the other inhibitors of crystallization, magnesium also seems to reduce lithogenic risk factors (mainly oxaluria) [19]. The protective effect of magnesium in therapy appears to be effective when given in combination with potassium citrate. On the other hand, the low sulphaturia seems to correlate with the low protein intake of the patients observed in this series. Furthermore, it is described that the urinary pH can influence the inducers of crystallization. Thus, lowering urinary pH promotes crystallization of uric acid, its rise facilitates phosphate precipitation, while the degree of supersaturation of calcium oxalate is practically independent of pH [5]. In this study, all patients with struvite stones had a urinary pH between 6 and 7. And, their urinary pH was higher compared to that of patients with calcium oxalate stones. The mean (SD) urine density in this study was 1.018 (0.007). This suggests insufficient nocturnal diuresis. Thus, lithiasis patients should be encouraged to balance water intake during the day by promoting intake at bedtime or when waking up at night [2]. In addition, 23.3% of patients in this study had proteinuria, the mean protein concentration in the urine was above normal (i.e., 0.25 ± 0.49 g/L) and 11% of patients (mainly with lithiasis of the upper apparatus) had impaired renal function. Concentration hyperoxaluria was found in 11% of patients in this study. While Yang *et al*. [3] found hyperoxaluria only in 5% of cases.

This difference in the frequency of hyperoxaluria between these two studies should prompt further research (either by crystalluria or stone morphology) for lithogenic genetic diseases such as primary hyperoxaluria in Congolese patients. It should be noted in this regard that the prevalence of homozygous primary hyperoxaluria is significantly higher in North Africa compared to industrialized countries [20,21]. Consanguinity, which remains very widespread in certain regions of the DRC, might explain these findings. Hypercalciuria was present in this study in 26% of cases with a higher urinary calcium / creatinine ratio in calcium oxalate stones than in struvite. In China, hypercalciuria was found in 18.8% of lithiasis patients under the age of 18. In another report, hypercalciuria was found in 25 to 60% of calcium lithiasis patients [17]. We think that the small number of stones analyzed in this study and the heterogeneity of stones might have influenced the lack of association between urinary metabolic abnormalities and the majority bodies identified. In addition, the ammonium urate and majority carbapatite stones in this study were all infection stones (given their overall composition), thus justifying, like struvite, their association with proteinuria and hypocitraturia. This study has limitations that should be considered in interpreting these results: the small sample size and the heterogeneity of the different samples. Therefore, some important urinary parameters such as creatinine, and sodium were not considered. In this context, a nutritional survey among Congolese with lithiasis would be informative. In addition, crystalluria (an interesting exploration to establish the diagnosis of certain lithogenic metabolic diseases [22] could not be carried out in this case study given the treatment and long shelf life of the samples. Nonetheless, this is the first study describing the prevalence and the main urinary metabolic abnormalities that may be involved in lithogenesis in the DRC.

## Conclusion

Metabolic abnormalities are frequent in patients with lithiasis. Inadequate diuresis and hypocitraturia were the two lithogenic factors most often observed in this study. These results warrant implementation of metabolic evaluation as a standard of care, mostly in patients with lithiasis in order to decrease recurrence rate through specific treatments and preventive measures such as modification lifestyle and dietary habits.

### What is known about this topic

- The urinary metabolic abnormalities play an essential role in the etiological development of urolithiasis disease;
- The urinary metabolic abnormalities are involved in preventing recurrence of urolithiases disease;
- However, these various metabolic abnormalities and their frequency are not known due to a lack of studies in the DRC.

### What this study adds

- This study provides essential information on the frequency of urinary metabolic abnormalities in Congolese urolithiasis patients;
- It thus identifies the main urinary metabolic abnormalities that can influence lithogenesis in our study population;
- The study gives in passing on a small sample an idea about the major constituents of urinary stones in our regions.

## References

[1] Abadie-Cathala N, Amiel J, Conort P, Daudon M (1996). Bilan métabolique d´une lithiase urinaire en pratique courante, Travail commun des néphrologues et urologues du Comité de la Lithiase de l´Association Française d´Urologie (CLAFU). Progrès en Urologie.

[2] Traxer O, Lechevallier E, Saussine C (2008). Bilan métabolique d'un patient lithiasique, Le rôle de l'urologue. Prog Urol.

[3] Yang D, Tiselius HG, Lan C, Dong Chen, Kang Chen, Lili O (2017). Metabolic disturbances in Chinese children with urolithiasis: a single center report. Urolithiasis.

[4] Siener R, Hesse A (2003). Fluid intake and epidemiology of urolithiasis. Eur J Clin Nutr.

[5] Jungers P, Daudon M, Le Duc A (1989). Lithiase urinaire. Paris, flammarion.

[6] Kovacevic L, Wolfe-Christensen C, Edwards L, Sadaps M, Lakshmanan Y (2012). From hypercalciuria to hypocitraturia a shifting trend in pediatric urolithiasis?. J Urol.

[7] Doremus RH, Teich S, Silvis PX (1978). Crystallization of calcium oxalate from synthetic urine. Invest Urol.

[8] Fleisch H, Bisaz S (1962). Isolation from urine of pyrophosphate, calcification inhibitor. Am J Physioli.

[9] Smith LH, Meyer JL (1975). Urinary inhibitors of calcium oxalate crystal growth, Colloquium on renal lithiasis. Universyty Presses of Florida, Gainsville.

[10] Pyrah LN (1957). Renal calculus. Br J Clin Pract.

[11] Robertson WG, Peacock M (1972). Calcium oxalate crystalluria and inhibitors of crystallization in recurrent renal stone formers. Clin Sci.

[12] Nakagawa Y, Abram V, kezdy FJ, Kaiser ET, Coe FL (1983). Purification and characterization of the principal inhibitor of calcium oxalate monohydrate crystal growth in human urine. J Biol Chem.

[13] Gambaro G, Baggio B, Favaro S, Cicerello E, Marchini F, Borsatti A (1984). Rôle de la mucoprotéine de Tamm-Horsfall dans la lithogénèse oxalo-calcique. Nephrol.

[14] Scurr DS, Latif AB, Sergeant V, Robertson WG (1983). Polyanionic inhnibitor of calcium oxalate crystal agglomeration in urine. Proc Eur Dial Transplant Assoc.

[15] Wattanachai U, Thanida C, Thasinas D, Chanchai B, Phisit P, Kriang T (2019). Lime powder regimen supplement alleviates urinary etabolic abnormalities in Urolithiasis patients. Nephrology (Carlton).

[16] Daudon M (2005). Épidémiologie actuelle de la lithiase rénale en France. Ann Urol (Paris).

[17] Ernandez T, Chopard C S, Bonny O (2013). Approche pratique de la lithiase rénale: duo entre généralistes et spécialistes. Rev Med Suisse.

[18] Sakhaee K, Maalouf NM, Sinnott B (2012). Clinical review Kidney stones 2012: pathogenesis, diagnosis, and management. J Clin Endocrinol Metab.

[19] Azarfar A, Esmaeili M, Tousi N, Naseri M, Ghane F, Ravanshad Y (2016). Evaluation of the effects of magnesium supplement in primary and secondary preventions of nephrolithiasis: a systematic review. Reviews in Clinical Medicine.

[20] Daudon M, Bounxouei B, Santa Cruz F, Leite Da Silva S, Diouf B, Angwafoo III FF (2004). Composition des calculs observés aujourd'hui dans les pays non industrialisés. Prog Urol.

[21] Barsoum RS (2003). End-stage renal disease in North Africa. Kidney Int Suppl.

[22] Lemaire M (2018). La lithiase rénale: comment éviter la récidive?. Louvain Med.

